# Design of Multi-Specificity in Protein Interfaces

**DOI:** 10.1371/journal.pcbi.0030164

**Published:** 2007-08-24

**Authors:** Elisabeth L Humphris, Tanja Kortemme

**Affiliations:** 1 Graduate Group in Biophysics, University of California San Francisco, San Francisco, California, United States of America; 2 California Institute for Quantitative Biosciences, University of California San Francisco, San Francisco, California, United States of America; 3 Department of Biopharmaceutical Sciences, University of California San Francisco, San Francisco, California, United States of America; Columbia University, United States of America

## Abstract

Interactions in protein networks may place constraints on protein interface sequences to maintain correct and avoid unwanted interactions. Here we describe a “multi-constraint” protein design protocol to predict sequences optimized for multiple criteria, such as maintaining sets of interactions, and apply it to characterize the mechanism and extent to which 20 multi-specific proteins are constrained by binding to multiple partners. We find that multi-specific binding is accommodated by at least two distinct patterns. In the simplest case, all partners share key interactions, and sequences optimized for binding to either single or multiple partners recover only a subset of native amino acid residues as optimal. More interestingly, for signaling interfaces functioning as network “hubs,” we identify a different, “multi-faceted” mode, where each binding partner prefers its own subset of wild-type residues within the promiscuous binding site. Here, integration of preferences across all partners results in sequences much more “native-like” than seen in optimization for any single binding partner alone, suggesting these interfaces are substantially optimized for multi-specificity. The two strategies make distinct predictions for interface evolution and design. Shared interfaces may be better small molecule targets, whereas multi-faceted interactions may be more “designable” for altered specificity patterns. The computational methodology presented here is generalizable for examining how naturally occurring protein sequences have been selected to satisfy a variety of positive and negative constraints, as well as for rationally designing proteins to have desired patterns of altered specificity.

## Author Summary

Computational methods have recently led to remarkable successes in the design of molecules with novel functions. These approaches offer great promise for creating highly selective molecules to accurately control biological processes. However, to reach these goals modeling procedures are needed that are able to define the optimal “fitness” of a protein to function correctly within complex biological networks and in the context of many possible interaction partners. To make progress toward these goals, we describe a computational design procedure that predicts protein sequences optimized to bind not only to a single protein but also to a set of target interaction partners. Application of the method to characterize “hub” proteins in cellular interaction networks gives insights into the mechanisms nature has used to tune protein surfaces to recognize multiple correct partner proteins. Our study also provides a starting point to engineer designer molecules that could modulate or replace naturally occurring protein interaction networks to combat misregulation in disease or to build new sets of protein interactions for synthetic biology.

## Introduction

Proteins have evolved to operate within the context of crowded cellular milieus and complex functional networks [1]. It is not well understood how and to what extent protein sequences and structures are optimized for multiple and likely interdependent properties such as stability and efficiency of folding, low propensity for aggregation, and functional characteristics. Protein–protein interaction networks may impose particular pressures on amino acid residues in protein interfaces if each protein needs to maintain correct and avoid unwanted interactions. Not only specificity of interactions but also a defined level of promiscuity may be required, as it is known that many proteins use regions of overlapping interface residues to bind several partners at different points in time [2].

Protein design predictions offer great promise to help dissect the structural determinants of the interplay between promiscuity and specificity, as well as to create new molecules that interfere with defined cellular protein–protein interactions with high fidelity and selectivity [3]. Protein design methodologies have generally focused on choosing an amino acid sequence optimal for a specific criterion, such as protein stability or interaction energy with a single binding partner. These computational design techniques have led to several accomplishments, including the pioneering design of a complete protein [4], of novel protein folds [5,6], the engineering of catalytic activity into an uncatalytic scaffold [7,8], and the redesign of protein–protein interfaces [9–12]. Yet, sequences completely redesigned on a known protein fold often differ substantially from naturally occurring sequences [4], and the properties of designed proteins can be unusual. Top7, a computationally designed protein with a fold not previously seen in nature [6], has a complex folding landscape strikingly different from that of evolved small, single domain proteins [13]. Thus, if we wish to rationally design new proteins that can be expressed and function correctly in a cellular environment and in the context of many possible interaction partners, it is likely that we will need modeling procedures that are able to consider a variety of requirements defining optimal protein “fitness.”

Here we focus on the multiple constraints interaction networks may impose on protein interfaces, both to characterize the evolutionary and biophysical principles shaping these networks, and to develop computational design methods to reengineer them. Previous studies have suggested the importance of negative selection to maintain specificity for a single binding partner [14]. Havranek and Harbury developed a negative design strategy selecting against unwanted partners to predict highly specific coiled-coil interfaces [11]. We extend the idea of incorporating additional selection constraints into computational protein design by examining the inverse problem: how are multiple positive criteria, such as the binding of different partners, accommodated at multi-specific (e.g., promiscuous) protein interfaces?

We perform two computational experiments on 20 multi-specific proteins. First, we optimize each multi-specific interface sequence to maintain interactions with all known structurally characterized partners (multi-constraint protocol). Second, we predict interface sequences optimal for interacting with each partner individually (single-constraint protocol). We hypothesize that, to the extent that a multi-constraint protocol is a good approximation of pressures acting on promiscuous interfaces, predicted sequences should be more “native-like” when all characterized binding partners are included in the optimization procedure than if only the interaction with a single binding partner is considered. Further, we can compare the differences in interface sequences selected by each partner alone (single-constraint) and all partners considered together (multi-constraint). If multiple pressures play a role for sequence choices, this comparison should highlight which amino acids are compromises among the various outcomes favored by each binding partner individually.

We show that, overall, inclusion of multiple binding partners during optimization returns sequences closer to those found in native promiscuous interfaces. We find native interface residues predicted to be “hotspots” for each partner remain optimal in the context of optimization for single or multiple partners, while other positions may or may not undergo compromises in order to maintain binding of all partners. These trends resulted in the classification of two broad groups of multi-specific interfaces. In the first group, the number of native residues recovered as optimal was similar for optimizations performed over single or multiple partners. Here, key interactions within the interface appeared to be shared, and there was little evidence of compromise in binding preferences among all partners. In contrast, a second group of multi-specific proteins, including “hubs” such as small GTPases, ubiquitin, and actin, appeared to have optimized large fractions of their interfaces for binding multiple partners. In these cases, each partner appears to pick and choose subsets of the interface to make key interactions with, and integrating differences in the binding preferences over all partners often resulted in the native residues being the “optimal compromise” for maintaining binding of all partners.

Our method thus both predicts interface sites responsible for multi-specificity and provides an estimate of the magnitude of pressure exerted on sites by each interaction partner. The method we present here can be used as a predictive tool to study how naturally occurring amino acid sequences might have been constrained by any number of positive or negative criteria—including the ability to adopt two different conformations [15]—or as a protein design tool to rationally redesign variants of native proteins to have a desired set of properties matching user-defined constraints.

## Results

### Rationale: Test for Optimization and Compromise by Applying Multi- and Single-Constraint Design to Promiscuous Protein Interfaces

**Figure 1:**
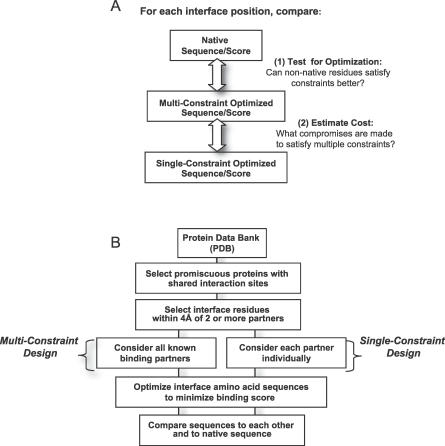
Computational Strategy and Methodology Flowchart (A) Computational strategy for determining the degree of optimization and predicted cost of multi-specificity. (B) Flowchart illustrating the methodology for generating a dataset of multi-specific proteins and computational protocol for predicting sequences optimal for each binding interaction alone (single-constraint) as well as sequences predicted to satisfying binding in the context of all structurally characterized partners (multi-constraint).

We set out to address two main questions (see Figure 1A). First, how optimized are native multi-specific interface sequences for binding multiple partners? It is known that the free energy of binding a single interacting partner is generally not evenly distributed among the native interface residues, but rather some hotspot positions are energetically more important than others [16]. Further, phage display experiments have revealed that substantial sequence plasticity may be tolerated at protein interfaces without significantly destabilizing, and often improving, binding of a single partner [17–19]. Thus, only a subset of a protein sequence may need to be constrained to native in order for a single criterion to be satisfied, while other sequence positions may be less optimized and tolerate a wider set of amino acid types. We hypothesize that the presence of multiple constraints (e.g., multiple binding partners) might substantially restrict interface sequence space such that only native or near native amino acid residues would be tolerated at most sites in multi-specific interfaces. If this is true, sequences that have been computationally designed to optimize binding with all known interaction partners should be more “native-like” than sequences designed to bind each partner independently. Thus, for each promiscuous protein we examine, we compare the sequence predicted to be optimal by our multi-constraint protocol to the wild-type sequence in order to provide an estimate of how extensively each interface is optimized for multi-specificity (see Test for Optimization in Figure 1A1). Importantly, differences between predicted and wild-type sequences could highlight that evolved sequences are not necessarily optimized for maximal affinity but that other pressures are at play.

Second, we ask if each binding partner prefers similar interactions throughout the binding site, or if some partners need to make energetic compromises in order for multi-specificity to be maintained. To address this question, we compare sequences computationally designed to bind only one partner at a time (without consideration of the other characterized partners) with the sequence selected as optimal for interacting with all partners (see Estimate Cost in Figure 1A2). We reasoned that for a given interface position, if an identical amino acid is chosen when each partner is optimized separately as is selected when all partners are included in the optimization process, key interactions at that site might be highly shared among all partners. In contrast, some single-constraint optimizations might choose an amino acid type different from the one selected in the multi-constraint protocol. For such positions, we can use our scoring function to estimate the degree of compromise occurring between differing preferences seen among the interaction partners.

We imagined two extreme case scenarios. If all binding partners of a given promiscuous protein prefer similar interface sequences (“shared” scenario), single- and multi-constraint optimizations are expected to give similar results and comparable agreement with wild-type sequences (termed “native sequence recovery”). If only a core set of shared residues is sufficient for binding to all partners, the total native sequence recovery over the entire interface could be low, as the exact amino acid identity of peripheral residues may be less important. Alternatively, each residue in a multi-specific interface could be optimal for only one or few partners (“multi-faceted” scenario). In this case, designed sequences from single-constraint simulations would be expected to resemble the wild-type sequence only for certain positions, and these positions could be different for each partner. The multi-constraint simulations should act to integrate preferences across all partners and would be expected to generate sequences that are more native-like than those resulting from optimization for any single binding partner alone. For this scenario, there could be significant tradeoff between the preferences of differing partners, and amino acid residues within this class of interfaces could be compromises with respect to the amino acid type preferred by some or all partners. However, for each interface position, we hypothesize that there should be an “optimal compromise” that satisfies the constraints imposed by all partners to maintain multi-specific binding.

### Computational Strategy

**Figure 2:**
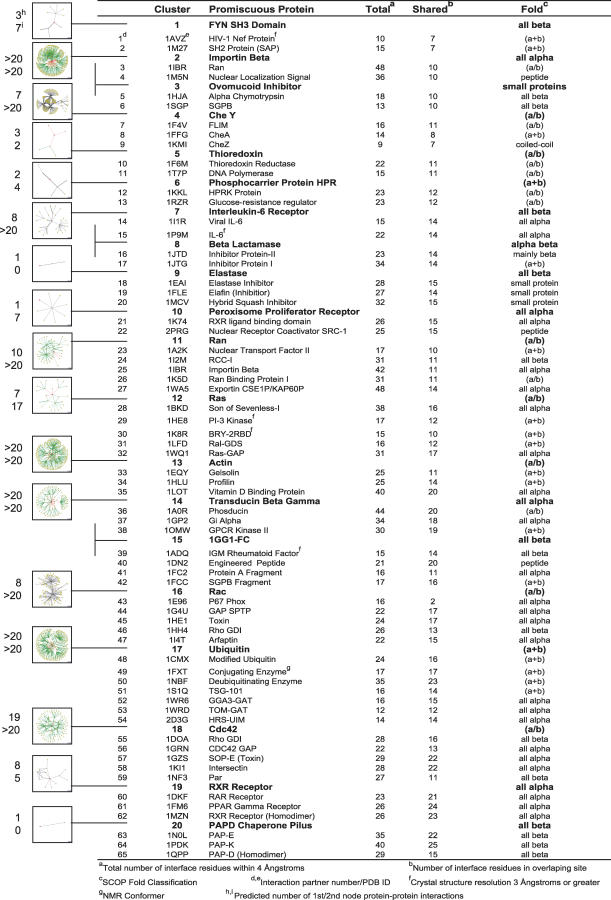
Dataset of Promiscuous Proteins PDB codes and descriptions of 20 promiscuous proteins and their 65 crystallized interaction partners. For each binding partner, the total number of residues it contacts (within 4 Å) on its promiscuous binding protein as well as the number of these residues which are also utilized by at least one other characterized binding partner are given in the “Total” and “Shared” columns. Fold classes are as assigned using SCOP [20]. Protein–protein interaction maps of sequence homologs to the promiscuous proteins in our dataset (see Methods) are as taken directly from the Database of Interacting Proteins [21], http://dip.doe-mbi.ucla.edu/, see Table S21). Root nodes are colored red and the number of first (orange) and second (yellow) shell nodes for each map is given on the far left. Edges are color-coded based on the reliability of data used to infer interactions, with green lines indicating data verified by one or more computational methods and red lines depicting unverified high-throughput screens. The width of lines in interaction graphs reflects the number of independent experiments verifying each predicted interaction.

Our computational protocol to test for optimization and compromise in multi-specific interfaces outlined above is illustrated schematically in Figure 1B. To determine whether the shared or multi-faceted strategies are used in naturally occurring promiscuous interfaces, we first compiled a dataset of protein complexes from the PDB (Protein Data Bank) (see Methods). Each promiscuous protein along with all its structurally characterized binding partners is listed in Figure 2. In total, we examined 65 PDB complexes, each of which included one of 20 multi-specific proteins. While this analysis is inherently limited by the set of promiscuous proteins characterized in the PDB and ignores much known information on biological interactions, it has the advantage that we can rely on high-resolution structural information for each of the complexes, and hence are more likely to obtain reliable predictions from protein design methods. Our dataset of 20 promiscuous proteins is nevertheless quite broad and includes all SCOP (Structural Classification of Proteins) [20] classes (except membrane proteins) as well as representatives from diverse functional families such as signaling proteins (GTPases, CheY), structural proteins (actin), ubiquitin, and several enzymes (see Figure 2). Further, in order to estimate the connectivity or number of putative protein–protein interactions for each promiscuous protein in our dataset, we performed a BLAST search against sequences within the Database of Interacting Proteins (DIP, http://dip.doe-mbi.ucla.edu/ [21]). Protein–protein interaction graphs for homologs to the multi-specific proteins in our set (leftmost column of Figure 2; e-value < 1 * 10^−9^, see Methods) suggest that at least half of the proteins we analyze can be classified as “hubs” (first shell nodes > 5; second shell nodes > 15) and that many of these proteins are involved in cellular signaling processes.

As we wished to examine promiscuous interface positions believed to be under multiple constraints, only interface positions that had an atom within 4 Å of two or more separate binding partners were considered in our analysis. On average, each characterized binding partner contacted 15 (±4.5) residues in this overlapping set (see Figure 2). Any conformational changes occurring between the different complexes were taken into account implicitly by using the backbone conformations directly from each complex PDB structure.

All computational protein design experiments used RosettaInterface and RosettaDesign, which have previously been used to predict binding energy hotspots in protein–protein complexes and to reengineer specificity in protein interfaces [12,22]. The scoring function [6,22] is dominated by atomic packing interactions, an orientation-dependent hydrogen potential [23], and an implicit solvation model [24]. Side chain rotamers were modeled on a fixed backbone, and optimal rotameric conformations were chosen for each complex backbone using a Monte-Carlo simulated annealing protocol. Sequence optimizations used a genetic algorithm [11], and fitness for binding was evaluated using inter-molecular scores (see Methods). Single-constraint optimizations minimized the binding score for interaction with a single partner while multiple-constraint optimizations minimized the sum of the calculated binding scores over all partners (see Methods).

### An Example Case Study: Ran GTPase Shows Multi-Faceted Binding

**Figure 3:**
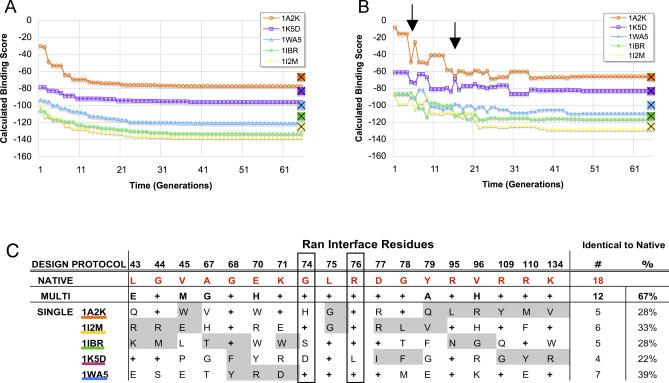
Single- and Multi-Constraint Simulation Trajectories and Sequences Selected for the Multi-Specific Protein Ran Trajectories of single-constraint (A) and multi-constraint (B) optimizations. PDB codes for all complexes with the five different binding partners are given in the legend. For reference, the score of the native amino acid sequence for each binding partner is marked on the *y*-axis (squares, final generation). Scores among partners are correlated for multi-constraint simulations (arrows). (C) Optimal interface sequences taken from the endpoint of the trajectories in (A) and (B). The first row in the table contains the interface residue PDB numbering, the second row lists the native sequence (red), and the following rows list sequences predicted to be optimal in each simulation: multi-constraint (second sequence), single-constraint (third through seventh sequences). Plus signs in the table denote that the wild-type amino acid residue type was recovered as optimal. The number and percent of interface residues recovered as identical to native is shown for each simulation in the rightmost column. Grey shading denotes interface positions not within 4 Å of the shaded interaction partner (see Methods).

Before discussing results over the entire dataset (complete data for all promiscuous proteins in our set are available as Tables S1–S20), we consider as a representative example the promiscuous protein Ran with five of its structurally characterized interaction partners (Figure 3). One multi-constraint and five single-constraint optimizations were performed for the Ran set. The trajectories of the five independent single-constraint optimizations monotonously decrease in score at each generation, and in each case the converged final sequence is predicted to have a binding score better than wild-type (Figure 3A, crosses at final generation (right edge of the graph)). Additionally, the sequences selected as optimal in each single-constraint simulation differ significantly from native (22%–39% native sequence recovery, plus signs in Figure 3C).

In contrast, the trajectories of the multi-constraint simulation show correlated changes in binding scores as each sampled sequence is evaluated separately in the context of the five complexes (Figure 3B). Cases where the simulation makes tradeoffs that are more favorable to some partners and less favorable for others can be clearly seen (arrows in Figure 3B). Here, the sum of scores over all complexes decreases with time and the final converged sequence ranks closer to the native score than the sequences selected by the single-constraint optimizations (compare endpoints of trajectories of Figure 3A and 3B with crosses at the final generation). Most notably, the amino acid sequence selected as optimal by the multi-constraint protocol is quite similar to the evolved wild-type sequence (67% identical to wild-type, plus signs in Figure 3C).

In the Ran example, the high native sequence recovery seen in the multi-constraint optimization indicates that a significant fraction of wild-type residues in this promiscuous interface is optimized for multi-specificity by “adding up” information from single-constraint optimizations (Figure 3C). This is consistent with the multi-faceted scenario described above. Further, the multi-constraint trajectories illustrate that there may be tradeoffs in preferences among the binding partners (Figure 3B, arrows), and comparison of sequences selected by the single- and multi-constraint simulations suggest interface positions where the wild-type residue may represent a compromise to allow promiscuity.

**Figure 4:**
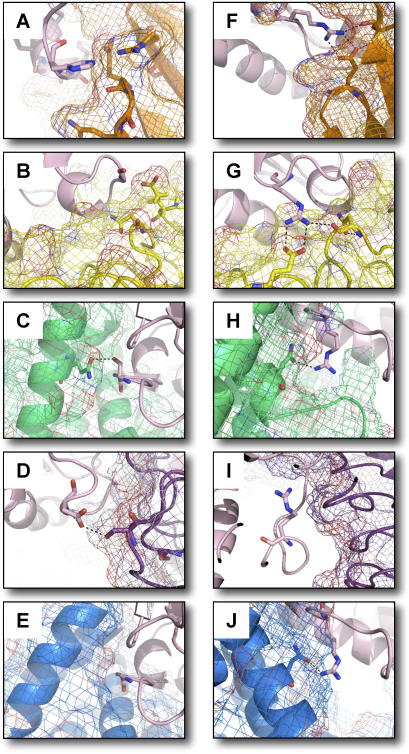
Single- and Multi-Constraint Models for Two Ran Interface Sites Shown are computational models of interface regions around residues predicted to be optimal for binding each partner (orange, 1A2K.pdb; yellow, 1I2M.pdb; green, 1IBR.pdb, purple, 1K5D.pdb; blue, 1WA5.pdb) of Ran (pink). Single-constraint predictions for residue 74 (A–E) (wild-type glycine) indicate compromise among the preferences of the five partners. Three partners (A,C,D), when optimized alone, prefer a residue with greater hydrogen bonding capabilities than the wild-type glycine. Steric constraints imposed by the remaining two partners (B,E) forced selection of the wild-type glycine by the multi-constraint protocol. Multi-constraint predictions for residue 76 are shown in panels F–J. The wild-type arginine is also chosen in all single-constraint predictions where it mediates an inter-chain hydrogen bonding network (F,G,H,J). Single-constraint selection of leucine at position 76 for 1K5D.pdb is not shown.

Figure 4A–4F depicts one such instance where several single-constraint optimizations select residues differing from native, yet the multi-constraint optimization integrates the single partner preferences to recover the wild type-glycine (single-constraint models shown in Figure 4A–4F are for the interface region around residue 74, first box in Figure 3C). The design simulations predict that three of Ran's binding partners (Figure 4A, 1A2K.pdb; Figure 4C, 1IBR.pdb; Figure 4D, 1K5D.pdb) prefer side-chains larger that the wild-type glycine that have additional side-chain hydrogen bonding capability. However, tight steric constraints for binding the remaining two partners (Figure 4B, 1I2M.pdb, and Figure 4E, 1WA5.pdb) necessitate glycine to be the “optimal” compromise for this interface position. Similar instances of compromise at interface positions that are under substantial steric constraint with a subset of the interaction partners are a common pattern in our dataset; many of these cases involve wild-type glycine residues.

In contrast to the compromised scenario described above, Figure 4G–4J (multi-constraint models shown in Figure 4G–4J are for the interface region around residue 76, second box in Figure 3C) depict a Ran interface residue that our simulations predict to be highly shared among all partners. Here the wild-type residue, arginine, is correctly recovered by every single-constraint simulation where it mediates an inter-chain hydrogen bonding network. This is the case for all partners except one (see Figure 4I). Here the interchain interactions are formed largely by the aliphatic part of the arginine side chain, and design simulations favor a leucine residue. Hence, for this interface position, where the multiple-constraint simulation also correctly selects the wild-type arginine, there is little indication that recovery of this native amino acid is the result of compromises among the interaction partners. Interestingly, the Ran interaction partners depicted in Figure 4F and 4G form very similar hydrogen bonding interactions with the wild-type arginine, although the partner proteins comprise different fold classes. This behavior of physicochemically similar interactions formed by structurally distinct interfaces has been observed previously [25,26].

### Sequences Selected by Multi-Constraint Simulations Can Be Substantially More Native-Like Than Single-Constraint Sequences

**Figure 5:**
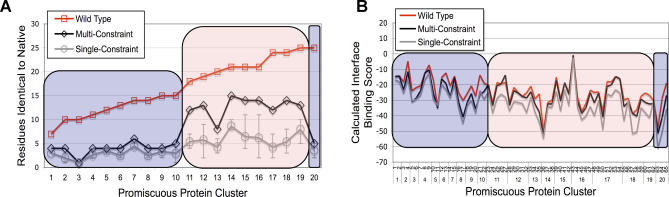
Comparison of Native Amino Acid Recovery and Predicted Binding Scores of Native, Single-Constraint, and Multi-Constraint Sequences (A) The number of residues recovered as identical to native are plotted for each promiscuous protein (see Figure 2). For reference, the size of the shared interface is shown for each protein in red. For roughly half the dataset, (group II, pink shading), sequence recovery from the multi-constraint simulations (black) significantly out-performed the average single-constraint recovery (grey). The remaining proteins (group I, blue shading) showed similar native recovery regardless of whether sequences were optimized with respect to one or all characterized partners. Error bars represent the best and worst native sequence recovery in a single-constraint optimization. (B) Calculated binding scores of native (red), single-constraint (grey), and multi-constraint (black) sequences for each of the 65 complexes examined in this study (see Figure 2). Sequences selected by single- and multi-constraint optimizations often show a favorable decrease in binding score relative to native sequences for group I proteins (blue shading), while multi-constraint binding scores were close to native for group II proteins (pink shading).

We next investigated whether the trend of optimization for promiscuity using the multi-faceted scenario we observed for Ran was common in our dataset. In total, 65 separate single-constraint optimizations and 20 multi-constraint optimizations were performed (Figure 2). Figure 5A shows that, over our entire dataset, sequences predicted as optimal by the multi-constraint protocol are more native-like than the sequences selected in the corresponding single-constraint runs (compare distance from red squares of black diamonds or of grey circles). There was only one instance (elastase complexed with inhibitors, promiscuous protein set #9) where the single-constraint optimization for binding one of the partners outperformed the multi-constraint protocol in native amino acid recovery.

Upon closer look at the pattern of interface residues recovered as native in each case, there seem to be two broad groups of multi-specific interfaces represented in the dataset. About half of the proteins comprised group I (blue shading, Figure 5A), for which the improvement in native sequence recovery in multi-constraint optimizations over single-constraint optimizations was small and total native amino acid recovery was low, regardless of interface size. As described for the shared scenario above, the low native sequence recovery could be due to all interaction partners binding via a few key residues, with the residues peripheral to these free to vary in sequence. This behavior is likely for several group I proteins including elastase, ovomucoid inhibitor, and the SH3 domain complexes. These proteins bind their targets within a narrow groove or cavity, and in addition a considerable fraction of interactions may be mediated through backbone contacts [27]. Low native sequence recovery in group I could also be influenced by inclusion of cross-species interactions (enzyme-inhibitor complexes and interleukin 6 receptor binding to mammalian and viral interleukin) as well as lack of sufficient constraints to fully specify the wild-type sequence (see discussion below).

In contrast, for the other half of the proteins in our dataset (group II), sequence optimization over all characterized binding partners resulted in significant improvements in native sequence recovery compared with optimizations for binding to a single partner (pink shading, Figure 5A). Here, as described for the multi-faceted scenario above, the multi-constraint optimization procedure was able to “add up” differing amino acid preferences among partners. The resulting high recovery of native amino acids indicates that binding interfaces for proteins in this group are optimized for multi-specificity. Additionally, as compared with group I, group II proteins tended to use larger and flatter interfaces to mediate binding, were more likely to show high connectivity in protein–protein interactions networks, and bound interaction partners with a greater number of different fold types (see Figure 2). Although generalizations of our conclusions are necessarily limited by the restricted size of our dataset of 20 proteins, a “multi-faceted” recognition pattern spread over a large interface may be a common strategy used by highly connected signaling hubs to bind diverse partners.

### Binding Scores of Sequences Selected by Multiple-Constraint Simulations Are Closer to Native Than Those of Single-Constraint Sequences for Group II Interfaces

We have shown that for about half the multi-specific proteins in our dataset (group II), the multi-constraint–designed sequences were substantially more native-like than single-constraint sequences (Figure 5A). According to our rationale outlined above, this suggested a significant level of optimization for multi-specificity in these interfaces. However, not all interface positions were predicted to be native-like, and native sequence recovery over the whole interface in multi-constraint simulations varied between 40% and 71% in this group.

Non-native amino acids could be chosen by our optimization protocol because they are predicted to be more favorable than the wild-type residue or, alternatively, because a number of different amino acid types are allowed at a certain position without substantial energetic differences. To test whether the non-native interface residues selected by the design simulations were predicted to lead to significant interface stabilization, we compared the binding scores of sequences selected by the single- and multi-constraint protocols with the scores of the wild-type sequences. For both group I and group II, optimization for only a single binding partner always resulted in a favorable decrease in predicted interface binding score (Figure 5B, grey line) relative to the score of the wild-type amino acid sequence (Figure 5B, red line). The binding score patterns for multi-constraint optimizations (Figure 5B, black line), however, differed among the two groups: multi-constraint binding scores were often similar to single-constraint scores for group I proteins (compare black and grey lines, blue shaded box), while for group II proteins multi-constraint binding scores were much closer to those calculated for the wild-type sequences (compare black and red lines, pink shaded box).

The division of our dataset into two groups suggested by the native sequence recovery results (Figure 5A) was thus mirrored in the predicted binding score patterns for wild-type and designed sequences (Figure 5B). Our simulations suggest that for group I proteins, where sequences and binding scores for single- and multi-constraint optimizations were similar, there might be non-native amino acids which could improve the promiscuous compromise and at the same time strengthen each interaction with each binding partner alone. In contrast, non-native amino acids selected for group II proteins in multi-constraint simulations are predicted to offer little improvement over the binding scores of the original wild-type sequences; this confirms our notion of high levels of optimization for multi-specificity in this group. Interestingly, while our simulations sought solely to maximize binding affinity for each partner, and did not explicitly consider either the relative binding affinities among partners or that naturally occurring interfaces often need to be transient, incorporation of multiple constraints alone was often sufficient to predict sequences with binding scores near or identical to that calculated for native sequences.

### For All Multi-Specific Interfaces, Energetically Important Residues Are Generally Optimized for Binding

We next investigated, on a per-residue basis, at which interface positions our optimization protocols predicted native residues to be suboptimal. Experimental analysis of residues critical for maintaining binding with respect to a single interaction partner have shown that often only a subset of the interface comprises key hotspot residues optimized for binding [16,17] and that other non-hotspot positions may show a high degree of plasticity [19]. We thus wished to examine how often native residues were being recovered as optimal by our single- and multi-constraint simulations at positions calculated to be energetically important hotspots.

For each binding partner, we calculated the per-residue score of the native residue at every interface position, and labeled sites with a native per-residue score of less than −2 as a predicted hotspot. Next we calculated for each position the difference in score between the residue selected by each of our protocols and the score of the native residue (see Test for Optimization in Figure 1A1). We reasoned that small score differences (<1 score units; scores are parameterized to approximate kcal/mol [22]) should reflect that a given optimization protocol recovered the native (or energetically similar to native) residue during optimization, and large score differences (>1 score units) should indicate the extent to which a non-native residue is predicted to improve binding affinity over native.

**Figure 6:**
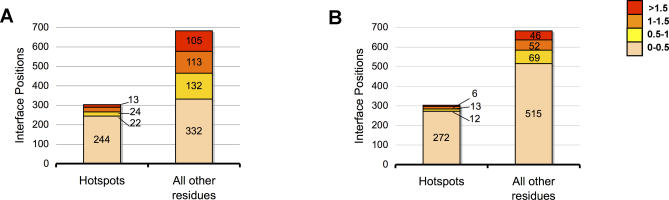
Distribution of Optimization in Promiscuous Interfaces Predicted per-residue binding score improvements (relative to native) for sequences selected in single-constraint (A) and multi-constraint (B) simulations. Coloring indicates the magnitude of predicted improvement over native. Darker-colored bars (compromise value 1–1.5, orange; more than 1.5, red) indicate positions for which the simulation predicts a non-native residue to bind stronger than native. Lighter-colored bars (compromise value 0–0.5, wheat; 0.5–1, yellow) indicate simulations recovered the native (or near-native) residue type. Whether optimization was in the context of single or multiple partners, positions calculated to be hotspots (see Methods) consistently returned the native amino acid as optimal (244/303 and 272/303, for single- and multi-constraint simulations, respectively). In contrast, roughly half of non-hotspot interface positions were predicted as suboptimal for binding when each partner was considered separately (350/682), but only a quarter (167/682) were estimated to still be suboptimal in the context of binding multiple partners. Overall, the total number of interface sites for which improvements in binding scores could be found was significantly less for multi-constraint optimizations. Scores for the same residue position with differing binding partners are included in all plots.

At hotspot positions, whether optimizations were performed with respect to single or multiple partners, native (or energetically equivalent) residues were recovered for each partner with high fidelity (Figure 6A and 6B, wheat bars, 244/303 and 272/303 for single- and multi-constraint optimizations, respectively). This inability to predict non-native residues scoring better than native at hotspot positions was seen for proteins in both group I and group II (see Figure S1). In contrast, at non-hotspot positions, the native residue was predicted to be suboptimal (yellow, orange, red bars) with respect to binding a single partner in approximately half of all instances (Figure 6A, “all other residues”, 350/682). This is in agreement with experimental phage display data showing the native residue to often be suboptimal for binding at non-hotspot positions [19]. When considered in the context of binding multiple partners, however, these same non-hotspot sites often are now predicted to be suboptimal in only 14% of all instances (“All other residues” in Figure 6B; yellow, orange, red bars 167/682). Thus, we find that the need to maintain multi-specificity imposes constraints primarily on non-hotspot residues. This results in native residues being recovered more often at such sites as they become the “optimal compromise” for binding of multiple partners. This trend for increased recovery of native residues at non-hotspot positions during multi-constraint simulations was much stronger for proteins in group II than in group I (see Figure S1).

### Distributions of Shared and Compromised Interactions in Promiscuous Interfaces

Finally, we wished to estimate the extent of compromise each multi-specific protein in our dataset made in order to maintain binding to all its partners compared with the “ideal” interaction it could have if only a single partner was considered (see the section Rationale, and Estimate of Cost in Figure 1A2). For each site within an interface, each partner was assigned a “compromise value” (ranging from 0 to 2). Compromise values were defined as the per-residue difference in score of the amino acid selected when each partner was optimized alone (single-constraint) and the residue selected at the same site when all partners were included in the optimization protocol (multi-constraint). The interface site itself was then assigned the largest compromise value seen among all binding partners. For each position in the interface, this number should provide a rough estimate of the maximal amount of tradeoff paid by any partner due to the necessity of other partners binding *via* the same site (see Methods and Figure 1A2). Small compromise values (0–1 score units) should indicate that all binding partners prefer the same (or similar) residue type as optimal, regardless of the presence or absence of other binding partners. Larger values (>1 score units) suggest that for at least one partner, a non-native amino acid is predicted to make more favorable interactions than the wild-type, but may not be tolerated when preferences of all additional binding partners are considered.

**Figure 7:**
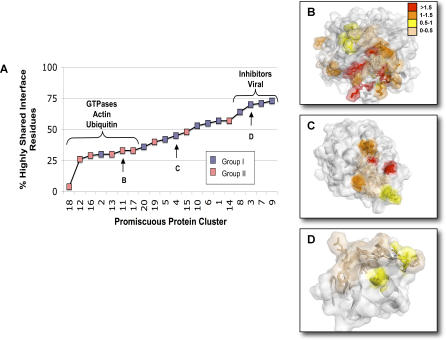
Distribution of Constraint Scores in Promiscuous Interfaces Tradeoff at each interface position in our dataset was estimated by the per-residue difference in scores of amino acids chosen when each partner was optimized alone as compared with when all binding partners were considered in the optimization procedure (see Figure 1A2). The percentage of interface sites displaying the lowest level (0–0.5) of “tradeoff value” (see Methods and text) is shown for all 20 proteins in our dataset (A). Such positions are predicted to be highly shared, in that no partner considered had to “give up” potential gain so that other partners could fulfill their optimal interactions. Blue and pink shading denotes whether each protein was assigned to group I or II. Right-hand panels show color-coded mappings of constraint scores onto three promiscuous protein interfaces calculated to display high (B) (Ran set #11), medium (C) (CheY set #4), and low (D) (Ovomucoid Inhibitor set #3) compromise. Compromise values are colored as follows: 0–0.5, wheat; 0.5–1 yellow; 1–1.5 orange; >1.5 red.

Figure 7A shows, over our entire dataset, the percentage of sites within each protein interface calculated to have a compromise score between 0 and 0.5. These positions are predicted to be essentially shared, in that no partner considered would have to give up potential gain so that other partners could fulfill their optimal interactions. While we observed a continuum ranging from interfaces calculated to have few completely shared interactions (all GTPases, actin, ubiquitin) to those for which the majority of interactions were shared (inhibitor complexes, SH3 domain), this analysis largely confirmed our earlier grouping of the multi-specific proteins within our dataset (Figure 7A, pink and blue boxes). A few group I proteins showed levels of compromise similar to that seen in group II. Interestingly, at least two of these proteins, importin beta (set #2) and cheY (set #4), were also calculated to be protein interaction “hubs” in our earlier analysis (see Figure 2). These proteins may thus also employ a “multi-faceted” binding strategy, and the low native sequence recovery seen with the multi-constraint protocol is likely due to our computational prediction being under-determined (since we lack structural information for a more complete set of binding partners). Likewise, we note that among the group II proteins, for IGG1-FC (set #15) many interactions were predicted to be shared by all binding partners, a result that is consistent with an earlier structural analysis of these proteins by Delano et al. [25].

To illustrate the three-dimensional distribution of predicted compromises in multi-specific interfaces, we generated color-coded mappings of compromise scores. Figure 7 shows representative maps for three promiscuous protein interfaces calculated to display high (Figure 7B, Ran), medium (Figure 7C, CheY), and low (Figure 7D, Ovomucoid Inhibitor) overall compromise (maps for the entire dataset are given in Figure S2). Throughout our dataset, higher compromise scores often occurred along the periphery of a binding site, while highly shared residues tended to be more centrally located. While further analysis is needed, this could indicate strong, shared interactions with core hotspots may be necessary for each partner to bind, but that it is along the rim of the overlapping interface site where compromises among the binding partners have to be integrated in order to maintain multi-specificity. This is reminiscent of the idea that hotspot residues necessary for binding often occur in interface cores sequestered from solvent, whereas other non-hotspot parts of the interface, possibly around the rim, account for recognition [16].

### Experimental Verification of a Non-Native Residue Predicted Optimal for Multi-Specific Binding

Our energetic analysis suggests that many positions within naturally occurring multi-specific interfaces have been optimized for binding to multiple partners, while some native amino acids are predicted to be sub-optimal in the context of single or even multiple partners. Over the entire dataset, the multi-constraint protocol recovered the native interface residue as optimal for just under half (161/338) of all interface residues examined. Ultimately, experimental data are needed to verify whether choices of non-native amino acids by our multi-constraint optimization protocol are incorrect predictions by our energy function, or whether the predicted choice would indeed strengthen binding for all partners.

In general, experimental data validating binding affinities of sequences predicted by our single- and multi-constraint simulations with all interaction partners were not available. However, we did observe one notable case where we could compare one of our predictions of an improved interface with direct experimental data. This occurred for the third domain of turkey ovomucoid inhibitor (set #3) at the key P1 position at which the inhibitor (or natural substrate) residue extends into a deep binding pocket. The predicted per-residue binding score at this site suggested that the wild-type residue was a hotspot crucial for maintaining binding with all partners, yet our multi-constraint protocol predicted a non-native amino acid residue to be significantly preferred over native by *all* partners. As discussed above, prediction of a native hotspot residue to be suboptimal was an infrequent occurrence throughout our dataset (see hotspots in Figure 6; yellow, red, orange bars).

Binding affinities for ovomucoid inhibitor mutants containing all 20 amino acids at the P1 position have been experimentally characterized for six different serine proteases [28]. This allowed us to compare the experimental preferences at the P1 position for the two serine proteases complexes in our dataset (chymotrypsin and SGPB, see Figure 2) with the computational predictions. The residue chosen at this site by the multi-constraint protocol, a phenylalanine, was ranked experimentally as the third and fourth most favorable residue for chymotrypsin and SGPB, respectively. There was no amino acid choice more favorable in common for both proteins and the native lysine residue was ranked eleventh and eighth, respectively. We note that while the multi-constraint protocol correctly selected the optimal choice for binding the two characterized binding partners in our dataset, other amino acid types may be optimal for selectively binding different combinations of the six serine proteases studied. Interestingly, the P1 residue of ovomucoid inhibitor is known to vary significantly in nature, with eight differing amino acid types occurring at this position in the 153 avian species analyzed [28].

## Discussion

Our study uses a protein design method that can in principle be applied to computationally select amino acid sequences under any set of positive and negative constraints that can be defined by a fitness function. Here we have made comparisons between single- and multi-constraint predicted and naturally occurring sequences to quantify optimization and compromise in multi-specific interfaces.

Our analysis indicates that first, the protocol presented here is able to detect optimization for multi-specificity in promiscuous interfaces, as sequences and binding scores from multi-constraint simulations are closer to native than those obtained in single-constraint optimizations. Second, we identify two distinct mechanisms for achieving multi-specificity: (1) shared or low compromise interfaces where a small subset of interface residues have been optimized such that all binding partners utilize this set as hotspots and (2) multi-faceted or intermediate compromise interfaces where a far larger percentage of the interface has been optimized for multi-specific binding and each partner picks and chooses a subset of interface residue interaction with which to make key interactions.

Signaling proteins with large, flat interfaces fall clearly within the “multi-faceted” group II, while enzymes, motif recognition domains, and receptors with smaller, narrower binding interfaces are often found within the shared group I. We speculate that the “multi-faceted” mode might have an evolutionary advantage for signaling interfaces, as here the chance that single mutations will deleteriously affect *all* binding interactions is reduced. On the other hand, a single mutation may substantially alter the *pattern* of interaction partners by now favoring certain interactions over others. In this way, multi-faceted interfaces may be more “evolvable” for new sets of interactions.

It is interesting to note that the ability to a priori predict binding sites from surface sequence conservation or surface cavity size has been shown to be easiest for proteins similar to those classified as “shared binding” by our methodology [29]. This is consistent with our observations, as in these cases there should be shared evolutionary pressure for conservation of key surface residues by all partners. In contrast, for proteins predicted to display some degree of compromise among the differing binding preferences of their multiple partners, evolutionary pressures could differ depending on which subset of binding partners is most strongly selected for over time. Further, allowing each partner to pick and choose its own subset of interface amino acids for key interactions, as in the multi-faceted case, could necessitate large, easily accessible (i.e., flat) binding surfaces with a certain degree of conformational flexibility; this mechanism could hence partly explain why flat surfaces and conformational variability are frequently seen in multi-specific signaling proteins such as G-proteins [30].

We hypothesize that there should be significant differences in the ease with which binding specificities among partners could be rationally modified and/or small molecule inhibitors could be designed for proteins exhibiting the two modes of multi-specificity described here. The patterns of varying amino acid preferences among different binding partners revealed by comparing the single- and multi-constraint protocols suggest mutations at specific interface positions that could rationally change the specificity or promiscuity seen among binding partners. However, these same factors might make drug or small molecule design toward “multi-faceted” interfaces more difficult. For the group II interfaces in our set, the different partners display varying interface residue preferences (see Figure 3C), and there may be a substantial number of constrained residues in each binding interface (see Figure 7). Hence, proteins using this mode of interaction may have fairly distributed hotspots that are difficult to interfere with by a small molecule targeted to a single region.

A caveat of our study is that first, generalizations may be somewhat limited because of the restricted size of our dataset of high-resolution structures. Second, the results presented here are necessarily dependent on the quality of the scoring function used for optimizations. However, improvement in native recovery seen in multi-constraint simulations could not be directly due to energy function biases, as the same scoring function was used for all simulations. The ability of the Rosetta scoring function to predict energetically important residues has been analyzed previously [22]. We note that amino acid types for which our simulations consistently select the native residue as the best (optimal) choice for binding multiple partners include tryptophan, tyrosine, and arginine (Figure S3; the predicted amino acid frequencies for W, Y, and R closely match the native distribution), amino acid types which have previously been shown to be energetically important in binding interfaces [16,31,32]. Interestingly, where allowed by steric constraints (for example at the interface periphery), we observed an increased selection in our simulations of larger amino acids such as tryptophan, arginine, and histidine, and against smaller amino acids such as alanine, threonine, and valine (Tables S1–S20 and Figure S3). While this could be due to approximations in our scoring function, an alternative explanation could be that these non-native sequences would, at least in some cases, truly bind more strongly. An overrepresentation of large hydrophobic residues may have been selected against in nature to maintain protein solubility in the absence of binding partners. In addition, while our computational protocol optimizes binding score, naturally occurring transient interfaces may not necessarily have evolved for strong binding. The complexes between small GTPases and their exchange factors (GEFs) may be examples of interactions that need to be transient to fulfill their cellular function: in the case of the ARF1-Sec7 interaction, the fungal metabolite Brefeldin A inhibits signaling by stabilizing the complex [33]. It may also be a general trend that multi-specificity must come at a cost of affinity [34]. Additional constraints not explicitly considered in our current protocol, such as selection at the level of on or off rates for complex formation could also account for differences in native and computationally selected sequences.

Lastly, we note that while the analysis presented here has focused on the ability of our simulations to identify the wild-type amino acid, strict conservation of a single native amino acid over evolutionary time is rare, and the tolerance for substitution to differing amino acid types can vary between sites in an interface [29]. For example, for the multi-specific protein Ras we found two instances (Table S12, positions 32Y and 67M) where we predicted the interface positions to be energetically important but failed to correctly recover the native amino acid. In both cases, the non-native amino acids selected by our multi-constraint simulations were among the evolutionarily tolerated set seen in a multiple-sequence alignment (unpublished data, generated as described in Methods). A clear extension of our method is thus not only to predict optimal but also a set of tolerated amino acid sequences for a given set of constraints (ELH and TK, unpublished data).

While we have applied the multi-constraint design protocol described in this work to examine whether and how promiscuous proteins are optimized for binding multiple partners, the methodology presented here is general and can be extended to analyze how any number of enumerable constraints (both positive and negative) affects sequence selection. A logical related analysis would be to characterize the sequence determinants of conformational flexibility where the input constraints would be stability for two or more different conformations. Further, the multi-constraint protocol introduced here is not only predictive of naturally occurring amino acid sequences, but also allows for rational redesign of proteins with altered binding properties which could be instrumental toward understand the role of specificity in protein interaction networks as well as in the engineering of biosensors and new cellular pathways.

## Methods

### Generation of a dataset of multi-specific proteins.

Each domain of every protein–protein interface listed in PIBASE (http://alto.compbio.ucsf.edu/pibase/ [35]) was classified using the standard SCOP domain definition. SCOP domains were clustered at 90% sequence identity. Clusters containing only intra-protein domain interactions (only one chain in the PDB file) were removed, and clusters with duplicates were merged, leaving 168 clusters. Additional filtering via PDB header descriptions to remove multi-subunit, viral coat, and immunoglobulins/MHC proteins resulted in approximately 50 clusters. All clusters containing multiple structures of the same promiscuous protein interacting with differing binding partners using an overlapping binding site (by visual inspection) were selected for the dataset of multi-specific proteins. Lower resolution structures of redundant protein–protein complexes were discarded, as well as all structures (except 1FXT) determined by NMR. PDB codes of the resulting 20 clusters are given in Figure 2.

### Energy function and preparation of structures.

All simulations were performed using the RosettaInterface and RosettaDesign methodologies as outlined in [6,22] and described below. The Rosetta scoring function is dominated by attractive and repulsive Lennard-Jones interactions, an orientation-dependent hydrogen bonding term [23], and an implicit solvation model [24]. Side chains from a rotamer library including the native amino acid PDB conformation and with additional rotamers around the χ1 and χ2 angles [4] were sampled on a fixed backbone using a Monte-Carlo simulated annealing optimization protocol.

All water molecules, heteroatoms, and hydrogens present in the original PDB were removed, and hydrogen atoms were added as previously described [23]. An initial round of side-chain Monte-Carlo minimization was then performed using the Rosetta scoring function, keeping all amino acid identities and backbone coordinates fixed, while selecting for the optimal rotamer at each side-chain position from the rotamer set as described above. After this initial minimization, all backbone and side-chain positions not determined to be in the shared interface were kept fixed for all subsequent steps.

### Single- and multi-constraint optimization protocol.

Amino acid positions on each promiscuous protein were considered for single- and multi-constraint design simulations only if any atom of two or more known binding partners was located within 4 Å of any atom of the side chain of interest. For promiscuous proteins with five or more characterized binding partners, only interface positions with an atom within 4 Å of three or more partners were considered. Each single- or multi-constraint optimization allowed all amino acids (except cysteine) to be substituted at each position examined. Positions for which the native residue was a cysteine were disregarded. For all simulations, a genetic algorithm was used to generate and propagate putative sequences based on inter-molecular scores, and optimal rotamers for each sequence were chosen separately with consideration of both inter- and intra-molecular interactions by simulated annealing Metropolis Monte Carlo for each fixed backbone as taken from the PDB. This ensured that in the multi-constraint protocol rotameric conformations could differ among binding partners even as identical interface amino acids were scored for each.

Simulations were started with an initial random population of 2,000 sequences, and the genetic algorithm was allowed to propagate for 100–200 generations. For single-constraint simulations, fitness was defined to be the inter-molecular score for a single complex while for multi-constraint simulations the fitness was a linear sum of the inter-molecular scores of a given amino acid sequence calculated across all characterized binding partners.

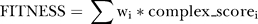



For all calculations, the weights (w_i_) were set uniformly to 1. For single-constraint simulations, the sequence that scored optimal with respect to a single complex independently was advanced to the next generation while the multi-constraint protocol advanced the sequence for which the fitness as defined above was minimized. Uniform crossover was used to generate the remaining sequences of the population for the following generation. Random mutation of any given interface sequence was allowed for each generation with a probability of 20% at any given interface position. Simulations converged (dependent on the size of the shared interface) on average within 50–130 generations (see Figure 3A).

### Per-residue energetic analysis.

Over the 20 multi-specific proteins in our dataset, 338 interface residues met the criteria for design. Consideration of each interface position in the context of the 65 characterized binding partners resulted in 1,199 individual interactions. For each individual interaction, a per-residue inter-chain score was calculated by summing, for any given residue on chain i, pair-wise contributions to the score from all residues on chain j ≠ i. An interface residue was classified as a hotspot for all binding partners for which the per-residue inter-chain score of the original native amino acid in the wild-type complex was calculated to be less than −2 (see pink shading, Tables S1–S20).

Estimates in predicted per-residue improvements (Figure 6) in binding affinity were made by calculating, for each binding partner, the difference in per-residue score of the amino acid chosen by single- or multi-constraint simulations (Figure 6A and 6B, respectively) from native. Positions for which the per-residue score for the native amino acid, as well as the amino acid chosen in single- and multi-constraint simulations was zero, were eliminated from the analysis. These 214 positions represented cases where one binding partner did not interact with an interface residue in contact with other partners in our dataset (see grey shading, Tables S1–S20).

Estimates of per-residue constraint (Figure 7) were made by calculating, for each binding partner, the difference in per-residue scores for the amino acid type/rotamer chosen in the single-constraint optimization from the respective score for the amino acid type/rotamer selected by the multi-constraint protocol. The largest magnitude of difference seen among all partners was the constraint value assigned. For simulations that did not recover the native amino acid type, constraint scores between sequences selected using single- and multi-constraint optimization were also calculated and assigned to the native amino acid type.

### Generation of protein–protein network graphs.

The complete sequence, as taken from the pdb files, of each promiscuous protein in our dataset was searched against all sequences contained within DIP (http://dip.doe-mbi.ucla.edu/ [21]). Hits were considered as significant if they had an e-value of less than 1*e^−9^. Protein–protein interaction graphs (Figure 2) were shown for sequences predicted to be homologous to Saccharomyces cerevisiae whenever possible. The DIP identification number, organism, e-value, and assigned DIP protein name for the interaction graph shown in Figure 2 are as given in Table S21.

### Multiple-sequence conservation of Ras.

A multiple-sequence alignment (MSA) and evolutionary rates for Ras were calculated using the automated Web server http://consurf-hssp.tau.ac.il for the Consurf-HSSP database [29] using the PDB ID code 1WQ1. Evolutionary conservation scores (1–10, 10 most conserved) were 9 and 8 for 32Y and 67M, respectively. 90% (186/206) of sequences within the multiple-sequence alignment for the native position 32Y contained either a Y or an H, while 89% (184/206) of sequences at the native position 67M contained H,I,L,M,Q, or V. Multi-constraint simulations selected 32H and 67H as optimal, respectively.

## Supporting Information

Figure S1 Group I and Group II Distributions of Optimization in Promiscuous Interfaces Predicted per-residue binding score improvements (relative to native) are shown for sequences selected in single-constraint (A,C) and multi-constraint (B,D) simulations for group I (top, pink shading) and group II (bottom, blue shading). Colored bars indicate the magnitude of predicted per-residue improvement over native. Darker-colored bars (compromise value >1, orange, red) indicate positions for which the simulation predicts a non-native residue to bind stronger than native. Lighter-colored bars (compromise value <1, wheat, yellow) indicate simulations recovered the native (or near-native) residue type. Group I and group II proteins show similar distributions of native residues predicted to be suboptimal when optimized for single binding interactions alone (A,C); compare red, orange, yellow bars. Optimization over multiple partners, however, differed between groups: a larger number of non-hotspot positions were still predicted to be suboptimal for group I when all partners were considered for optimization than seen in group II (“All other residues”; compare red, orange, yellow bars in (B,D)). This is consistent with our finding that native sequence recovery is lower overall for group I.
(206 KB PDF)

Figure S2 Distribution of Compromise for All 20 Promiscuous Proteins in the Dataset Constraint scores (see Methods) are mapped onto each promiscuous protein in the dataset. Darker colors indicate stronger tradeoff in that some partners considered are predicted to “give up” potential gain so that other partners could fulfill their optimal interactions. Overall, group I proteins (1–10, 20) display lower levels of tradeoff than seen in group II (11–19).
(5.1 MB PDF)

Figure S3 Amino Acid Frequency Distributions of Sequences Selected as Optimal in the Multi-Constraint Procedure For each amino acid type, the number of times an amino acid type was correctly recovered as native is shown as black striped bars. Non-native substitutions of each amino acid type are shown as white bars. The native amino acid distribution is plotted for reference (solid black line).
(216 KB PDF)

Table S1 Single- and Multi-Constraint Sequences Selected for FYN SH3 Domain For Tables S1–S20, the first row in each Table contains the interface residue PDB numbering, the second row lists the native sequence (red), and the following rows contain sequences predicted to be optimal in each simulation: multi-constraint (second sequence), single-constraint (third sequence to the last sequence). Plus signs denote that the native amino acid residue type was recovered as optimal. The number and percent of interface residues recovered to be identical to native is shown for each simulation in the rightmost column. Pink shading denotes that the original wild-type amino acid type was calculated to be a hotspot for the given binding partner, while grey shading signifies an interface position not within 4 Å of the respective interaction partner (see Methods).
(30 KB PDF)

Table S2 Single- and Multi-Constraint Sequences Selected for Importin Beta (31 KB PDF)

Table S3 Single- and Multi-Constraint Sequences Selected for Ovomucoid Inhibitor (31 KB PDF)

Table S4 Single- and Multi-Constraint Sequences Selected for Che Y (33 KB PDF)

Table S5 Single- and Multi-Constraint Sequences Selected for Phosphocarrier Protein HPR (31 KB PDF)

Table S6 Single- and Multi-Constraint Sequences Selected for Thioredoxin (32 KB PDF)

Table S7 Single- and Multi-Constraint Sequences Selected for Interleukin-6 (31 KB PDF)

Table S8 Single- and Multi-Constraint Sequences Selected for Beta Lactamase (31 KB PDF)

Table S9 Single- and Multi-Constraint Sequences Selected for Elastase (32 KB PDF)

Table S10 Single- and Multi-Constraint Sequences Selected for Peroxisome Proliferator Receptor (33 KB PDF)

Table S11 Single- and Multi-Constraint Sequences Selected for Ran (34 KB PDF)

Table S12 Single- and Multi-Constraint Sequences Selected for Ras (34 KB PDF)

Table S13 Single- and Multi-Constraint Sequences Selected for Actin (33 KB PDF)

Table S14 Single- and Multi-Constraint Sequences Selected for Transducin Beta Gamma (34 KB PDF)

Table S15 Single- and Multi-Constraint Sequences Selected for FC (34 KB PDF)

Table S16 Single- and Multi-Constraint Sequences Selected for Rac (34 KB PDF)

Table S17 Single- and Multi-Constraint Sequences Selected for Ubiquitin (37 KB PDF)

Table S18 Single- and Multi-Constraint Sequences Selected for CDC42 (35 KB PDF)

Table S19 Single- and Multi-Constraint Sequences Selected for RXR Receptor (34 KB PDF)

Table S20 Single- and Multi-Constraint Sequences Selected for PAPD (34 KB PDF)

Table S21 Source of High-Throughput Interaction Data for Promiscuous Proteins DIP (http://dip.doe-mbi.ucla.edu/) identification numbers, e-values, and protein names for sequences identified as homologs to the 20 promiscuous proteins in our dataset. Interaction graphs (see Figure 2) are taken directly from each DIP protein listed.
(57 KB PDF)

## Abbreviations

DIP: Database of Interacting Proteins
PDB: Protein Data Bank
SCOP: Structural Classification of Proteins
