# Supplementary material for: Design of Multi-Specificity in Protein Interfaces
Source: PLoS Comput Biol. 2007 Aug 24;3(8):e164. doi: 10.1371/journal.pcbi.0030164 (PMC1950952; doi:10.1371/journal.pcbi.0030164)
Supplement: Supplementary file 1 [file pcbi.0030164.sg001.pdf]

FIGURE S1

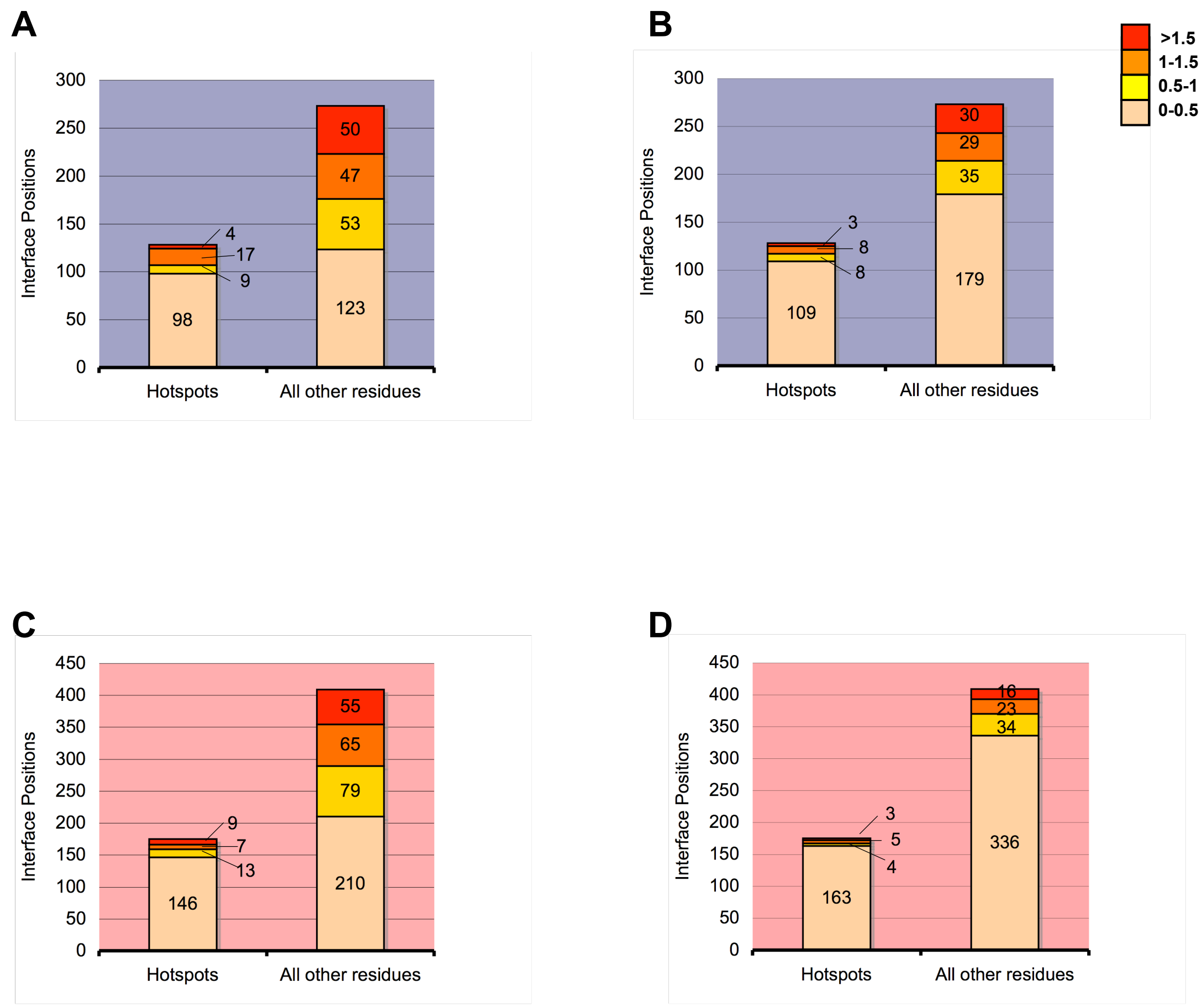

A
B
C
D
Interface Positions
Hotspots
All other residues
>1.5
1-1.5
0.5-1
0-0.5
98
9
17
4
123
53
47
50
109
8
8
3
179
35
29
30
146
13
7
9
210
79
65
55
163
4
5
3
336
34
23
16
