# Supplementary material for: Design of Multi-Specificity in Protein Interfaces
Source: PLoS Comput Biol. 2007 Aug 24;3(8):e164. doi: 10.1371/journal.pcbi.0030164 (PMC1950952; doi:10.1371/journal.pcbi.0030164)
Supplement: Supplementary file 3 [file pcbi.0030164.sg003.pdf]

FIGURE S3

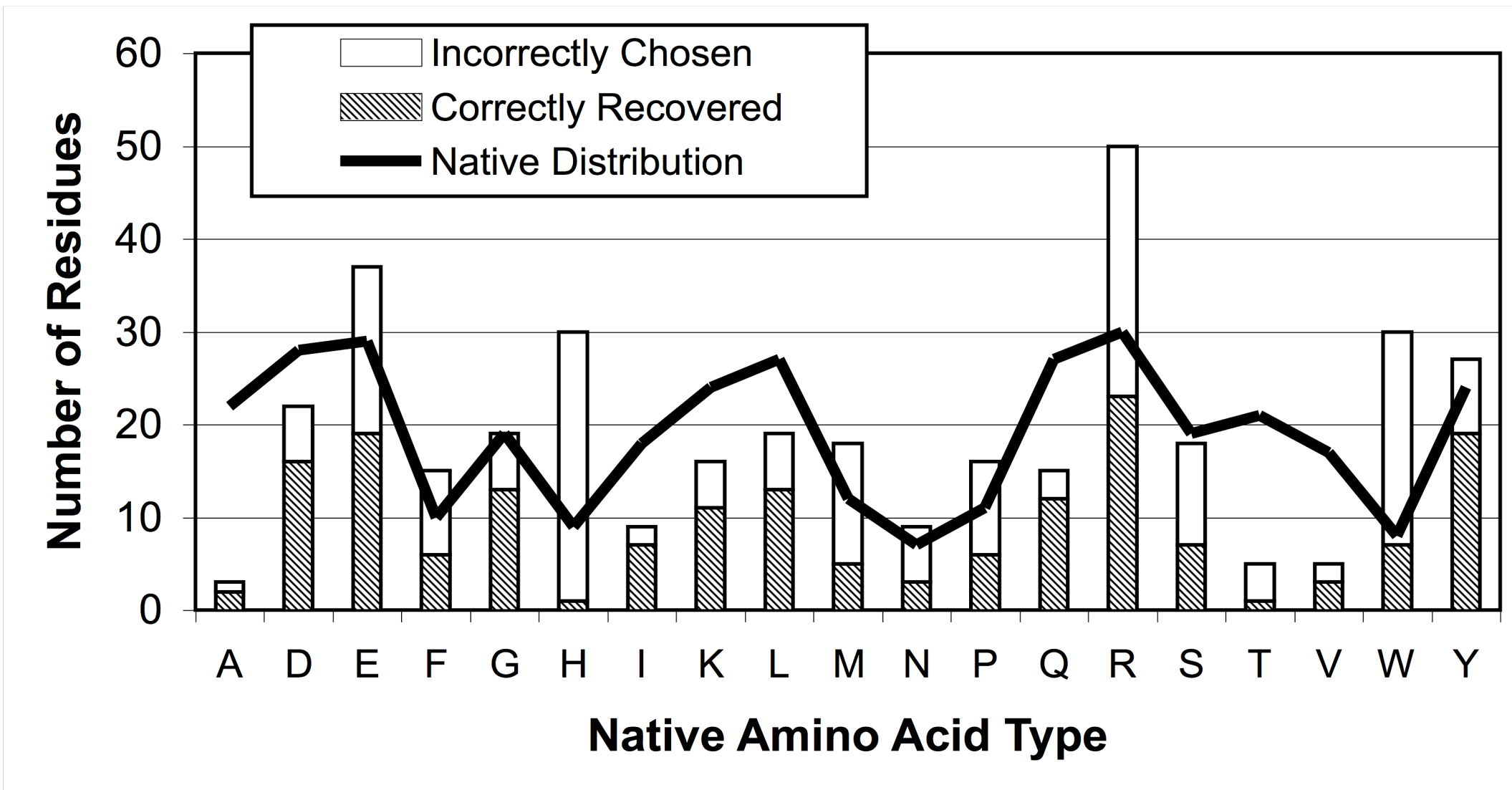

Incorrectly Chosen
Correctly Recovered
Native Distribution
60
50
40
30
20
10
0
Number of Residues
A D E F G H I K L M N P Q R S T V W Y
Native Amino Acid Type
