# Supplementary material for: Design of Multi-Specificity in Protein Interfaces
Source: PLoS Comput Biol. 2007 Aug 24;3(8):e164. doi: 10.1371/journal.pcbi.0030164 (PMC1950952; doi:10.1371/journal.pcbi.0030164)
Supplement: Supplementary file 4 [file pcbi.0030164.st001.pdf]

TABLE S1

Design Protocol	FYN SH3 Interface Residues							Identical to Native	
	93	96	97	98	99	100	119	#	%
NATIVE	Y	R	T	E	D	D	W	7	
**MULTI-**	**R**	**+**	**P**	**W**	**+**	**+**	**+**	**4**	57%
SINLGE- 1AVZ	R	+	S	Q	E	+	+	3	43%
1M27	R	E	+	W	+	E	+	3	43%
