# Supplementary material for: Design of Multi-Specificity in Protein Interfaces
Source: PLoS Comput Biol. 2007 Aug 24;3(8):e164. doi: 10.1371/journal.pcbi.0030164 (PMC1950952; doi:10.1371/journal.pcbi.0030164)
Supplement: Supplementary file 5 [file pcbi.0030164.st002.pdf]

TABLE S2

Design Protocol		Importin Beta Interface Residues										Identical to Native	
		55	106	156	159	239	274	278	281	288	342	#	%
NATIVE		V	P	Y	Q	Q	E	Q	E	D	W	10	
**MULTI-**		**L**	**K**	**+**	**E**	**+**	**W**	**+**	**+**	**Y**	**M**	**4**	**40%**
SINLGE-	1IBR	L	M	+	E	R	H	+	+	Y	+	4	40%
	1M5N	E	W	+	K	I	Y	W	Y	W	E	1	10%
