# Supplementary material for: Design of Multi-Specificity in Protein Interfaces
Source: PLoS Comput Biol. 2007 Aug 24;3(8):e164. doi: 10.1371/journal.pcbi.0030164 (PMC1950952; doi:10.1371/journal.pcbi.0030164)
Supplement: Supplementary file 6 [file pcbi.0030164.st003.pdf]

TABLE S3

Design Protocol		Ovomucoid Inhibitor Interface Residues										Identical to Native	
		13	14	15	17	18	19	20	21	33	36	#	%
NATIVE		K	P	A	T	K	E	Y	R	N	N	10	
**MULTI**		**W**	**W**	**P**	**E**	**F**	**H**	**W**	**+**	**I**	**V**	**1**	**10%**
SINGLE	1HJA	W	W	P	E	F	R	F	+	I	S	1	10%
	1SGP	W	W	R	E	Y	+	R	Q	E	R	1	10%
