# Supplementary material for: Design of Multi-Specificity in Protein Interfaces
Source: PLoS Comput Biol. 2007 Aug 24;3(8):e164. doi: 10.1371/journal.pcbi.0030164 (PMC1950952; doi:10.1371/journal.pcbi.0030164)
Supplement: Supplementary file 7 [file pcbi.0030164.st004.pdf]

TABLE S4

Design Protocol		CheY Interface Residues											Identical to Native	
		90	92	95	99	104	105	106	107	108	119	122	#	%
**NATIVE**		**A**	**K**	**I**	**A**	**S**	**G**	**Y**	**V**	**V**	**K**	**K**	**11**	
**MULTI**		**W**	**+**	**+**	**G**	**P**	**S**	**+**	**F**	**S**	**+**	**R**	**4**	**36%**
SINGLE	1F4V	R	R	+	W	N	S	H	W	S	+	I	2	18%
	1FFG	H	+	Q	+	P	H	+	W	F	W	+	4	36%
	1KMI	T	W	+	G	T	N	H	H	W	+	R	2	18%
