# Supplementary material for: Design of Multi-Specificity in Protein Interfaces
Source: PLoS Comput Biol. 2007 Aug 24;3(8):e164. doi: 10.1371/journal.pcbi.0030164 (PMC1950952; doi:10.1371/journal.pcbi.0030164)
Supplement: Supplementary file 8 [file pcbi.0030164.st005.pdf]

TABLE S5

Design Protocol		Phosphocarrier Protein HPR Interface Residues											Identical to Native	
		11	12	15	16	47	48	51	52	53	54	55	#	%
**NATIVE**		**D**	**S**	**H**	**A**	**I**	**M**	**M**	**S**	**L**	**G**	**I**	**11**	
**MULTI**		**R**	**R**	**+**	**W**	**H**	**+**	**F**	**+**	**H**	**+**	**F**	**4**	**36%**
SINGLE	1KKL	R	G	R	S	R	+	W	+	H	+	Y	3	27%
	1RZR	H	W	+	W	H	L	F	Q	W	+	E	2	18%
