# Supplementary material for: Design of Multi-Specificity in Protein Interfaces
Source: PLoS Comput Biol. 2007 Aug 24;3(8):e164. doi: 10.1371/journal.pcbi.0030164 (PMC1950952; doi:10.1371/journal.pcbi.0030164)
Supplement: Supplementary file 9 [file pcbi.0030164.st006.pdf]

TABLE S6

Design Protocol		Thioredoxin Interface Residues												Identical to Native	
		31	34	37	60	72	73	74	75	91	93	94	98	#	%
**NATIVE**		**W**	**P**	**M**	**I**	**I**	**R**	**G**	**I**	**V**	**A**	**L**	**Q**	**12**	
**MULTI**		**+**	**+**	**R**	**H**	**E**	**W**	**+**	**+**	**W**	**L**	**Q**	**W**	**4**	**33%**
SINGLE	1F6M	+	M	R	+	M	W	+	N	W	Q	W	R	3	25%
	1T7P	+	+	W	F	W	+	H	T	+	I	Q	W	4	33%
