# Supplementary material for: Design of Multi-Specificity in Protein Interfaces
Source: PLoS Comput Biol. 2007 Aug 24;3(8):e164. doi: 10.1371/journal.pcbi.0030164 (PMC1950952; doi:10.1371/journal.pcbi.0030164)
Supplement: Supplementary file 10 [file pcbi.0030164.st007.pdf]

TABLE S7

Design Protocol		IL-6 Interface Residues													Identical to Native	
		142	144	145	147	165	166	167	168	169	170	193	226	229	230	#	%
**NATIVE**		**W**	**T**	**H**	**F**	**S**	**T**	**V**	**Y**	**F**	**V**	**D**	**S**	**S**	**V**	**14**	
**MULTI**		**+**	**R**	**R**	**+**	**+**	**H**	**I**	**+**	**+**	**F**	**F**	**K**	**H**	**+**	**6**	**43%**
SINGLE	1I1R	+	R	R	+	+	E	I	+	+	F	R	K	H	D	5	36%
	1P9M	+	E	R	R	A	H	I	P	+	+	F	E	S	F	3	21%
