# Supplementary material for: Design of Multi-Specificity in Protein Interfaces
Source: PLoS Comput Biol. 2007 Aug 24;3(8):e164. doi: 10.1371/journal.pcbi.0030164 (PMC1950952; doi:10.1371/journal.pcbi.0030164)
Supplement: Supplementary file 11 [file pcbi.0030164.st008.pdf]

TABLE S8

Design Protocol	Beta Lactamase Interface Residues														Identical to Native	
	99	100	102	103	104	105	106	107	110	111	129	216	237	270	#	%
NATIVE	Q	N	L	V	E	Y	S	P	E	K	M/L	V	A	M	14	
**MULTI**	**H**	**W**	**R**	**E**	**+**	**+**	**G**	**H**	**+**	**+**	**Q**	**S**	**G**	**W**	**4**	**29%**
SINGLE 1JTD	+	W	H	H	P	W	L	H	+	+	Q	H	G	W	3	21%
1JTG	F	W	W	E	+	+	K	H	H	Q	Q	S	N	W	2	14%
