# Supplementary material for: Design of Multi-Specificity in Protein Interfaces
Source: PLoS Comput Biol. 2007 Aug 24;3(8):e164. doi: 10.1371/journal.pcbi.0030164 (PMC1950952; doi:10.1371/journal.pcbi.0030164)
Supplement: Supplementary file 12 [file pcbi.0030164.st009.pdf]

TABLE S9

Design Protocol		Elastase Interface Residues															Identical to Native	
		41	57	61	172	175	190	192	193	194	195	213	214	215	216	217	#	%
**NATIVE**		T	H	R	W	T	G	Q	G	D	S	T	S	F	V	S	15	
**MULTI**		**L**	**F**	**+**	**+**	**K**	**+**	**+**	**D**	**P**	**L**	**A**	**Y**	**Y**	**T**	**N**	**4**	**27%**
SINGLE	1EAI	L	F	V	+	K	+	+	N	P	I	M	K	W	T	W	3	20%
	1FLE	L	F	M	Y	W	Y	+	N	V	L	L	Y	+	I	W	2	13%
	1MCV	+	F	+	+	W	+	+	H	P	L	K	L	Y	A	P	5	33%
