# Supplementary material for: Design of Multi-Specificity in Protein Interfaces
Source: PLoS Comput Biol. 2007 Aug 24;3(8):e164. doi: 10.1371/journal.pcbi.0030164 (PMC1950952; doi:10.1371/journal.pcbi.0030164)
Supplement: Supplementary file 13 [file pcbi.0030164.st010.pdf]

TABLE S10

Design Protocol		Peroxisome Proliferator Receptor Interface Residues															Identical to Native	
		396	410	414	430	432	433	434	436	437	439	440	443	444	447	451	#	%
**NATIVE**		**D**	**Q**	**L**	**Q**	**F**	**A**	**K**	**L**	**Q**	**M**	**T**	**R**	**Q**	**T**	**Q**	**15**	
**MULTI**		**W**	**+**	**+**	**+**	**Y**	**+**	**R**	**H**	**E**	**K**	**E**	**P**	**W**	**H**	**+**	**5**	**33%**
SINGLE	1K74	W	M	+	K	R	P	W	Y	E	H	Y	D	W	W	N	1	7%
	2PRG	+	+	H	+	Y	+	W	H	W	K	D	F	E	Q	+	5	33%
