# Supplementary material for: Design of Multi-Specificity in Protein Interfaces
Source: PLoS Comput Biol. 2007 Aug 24;3(8):e164. doi: 10.1371/journal.pcbi.0030164 (PMC1950952; doi:10.1371/journal.pcbi.0030164)
Supplement: Supplementary file 14 [file pcbi.0030164.st011.pdf]

TABLE S11

Design Protocol		Ran Interface Residues																		Identical to Native	
		43	44	45	67	68	70	71	74	75	76	77	78	79	95	96	106	110	134	#	%
**NATIVE**		**L**	**G**	**V**	**A**	**G**	**E**	**K**	**G**	**L**	**R**	**D**	**G**	**Y**	**R**	**V**	**R**	**R**	**K**	**18**	
**MULTI**		**E**	**+**	**M**	**G**	**+**	**H**	**+**	**+**	**+**	**+**	**+**	**+**	**A**	**+**	**H**	**+**	**+**	**+**	**12**	**67%**
SINGLE	1A2K	Q	+	W	V	+	W	+	H	G	+	R	+	Q	L	R	Y	M	V	5	28%
	1I2M	R	R	E	H	+	R	E	+	G	+	R	L	V	+	H	+	F	+	6	33%
	1IBR	K	M	L	T	+	W	W	S	+	+	+	T	F	N	G	Q	+	W	5	28%
	1K5D	+	+	P	G	F	Y	R	D	+	L	I	F	G	+	R	G	Y	R	4	22%
	1WA5	E	S	E	T	Y	R	D	+	+	+	+	M	E	+	K	+	E	+	7	39%
