# Supplementary material for: Design of Multi-Specificity in Protein Interfaces
Source: PLoS Comput Biol. 2007 Aug 24;3(8):e164. doi: 10.1371/journal.pcbi.0030164 (PMC1950952; doi:10.1371/journal.pcbi.0030164)
Supplement: Supplementary file 15 [file pcbi.0030164.st012.pdf]

TABLE S12

Design Protocol		Ras Interface Residues																			Identical to Native	
		13	21	25	31	32	33	34	36	37	38	39	40	41	54	61	63	64	67	70	#	%
**NATIVE**		**G**	**I**	**Q**	**E**	**Y**	**D**	**P**	**I**	**E**	**D**	**S**	**Y**	**R**	**D**	**Q**	**E**	**Y**	**M**	**Q**	**19**	
**MULTI**		**D**	**L**	**R**	**R**	**H**	**+**	**+**	**+**	**+**	**+**	**+**	**+**	**+**	**+**	**+**	**+**	**+**	**H**	**+**	**13**	**68%**
SINGLE	1BKD	H	+	F	W	H	E	S	V	+	+	N	+	W	+	+	+	+	H	+	9	47%
	1HE8	L	M	R	R	W	E	K	R	+	P	+	H	W	I	G	W	H	W	K	2	11%
	1K8R	P	R	W	M	S	W	V	E	+	+	+	D	+	+	T	K	+	W	K	6	32%
	1LFD	+	F	R	R	R	+	N	W	+	E	R	W	M	W	E	W	T	M	R	3	16%
	1WQ1	D	R	R	+	H	+	+	+	M	+	H	S	W	W	+	+	+	W	W	8	42%
