# Supplementary material for: Design of Multi-Specificity in Protein Interfaces
Source: PLoS Comput Biol. 2007 Aug 24;3(8):e164. doi: 10.1371/journal.pcbi.0030164 (PMC1950952; doi:10.1371/journal.pcbi.0030164)
Supplement: Supplementary file 16 [file pcbi.0030164.st013.pdf]

TABLE S13

Design Protocol		Actin Interface Residues																			Identical to Native	
		113	143	144	145	146	147	148	166	167	169	173	286	287	288	290	345	355	371	372	373	#	%
NATIVE		K	Y	A	S	G	R	T	Y	E	Y	H	D	V	D	R	I	M	H	R	K	20	
**MULTI**		**R**	**+**	**S**	**R**	**+**	**+**	**S**	**+**	**H**	**+**	**E**	**+**	**H**	**W**	**+**	**+**	**W**	**R**	**N**	**N**	**8**	**40%**
SINGLE	1EQY	S	+	S	F	S	P	S	S	W	+	F	R	A	P	L	+	F	Q	E	W	3	15%
	1HLU	R	W	I	H	F	T	+	+	W	+	F	+	R	+	W	H	W	R	+	M	6	30%
	1LOT	R	F	L	R	+	+	M	+	H	Q	E	H	H	W	+	F	W	Q	N	H	4	20%
