# Supplementary material for: Design of Multi-Specificity in Protein Interfaces
Source: PLoS Comput Biol. 2007 Aug 24;3(8):e164. doi: 10.1371/journal.pcbi.0030164 (PMC1950952; doi:10.1371/journal.pcbi.0030164)
Supplement: Supplementary file 17 [file pcbi.0030164.st014.pdf]

TABLE S14

Design Protocol		Transducin Beta Gamma Interface Residues																					Identical to Native	
		55	57	59	75	76	78	98	99	101	117	119	144	145	186	188	228	230	246	290	314	332	#	%
**NATIVE**		**L**	**K**	**Y**	**Q**	**D**	**K**	**S**	**W**	**M**	**L**	**N**	**G**	**Y**	**D**	**M**	**D**	**N**	**D**	**D**	**R**	**W**	**21**	
**MULTI**		**R**	**+**	**+**	**+**	**R**	**H**	**+**	**+**	**+**	**+**	**+**	**P**	**+**	**+**	**+**	**+**	**D**	**+**	**N**	**+**	**+**	**15**	**71%**
SINGLE	1A0R	R	+	+	M	E	A	+	+	P	H	R	P	H	+	+	E	D	+	N	K	+	8	38%
	1GP2	W	+	F	+	Q	+	R	+	R	+	+	T	H	+	L	+	D	+	Y	S	+	10	48%
	1OMW	R	R	K	+	K	H	+	+	+	+	Y	D	W	R	+	E	E	W	Y	+	+	8	38%
