# Supplementary material for: Design of Multi-Specificity in Protein Interfaces
Source: PLoS Comput Biol. 2007 Aug 24;3(8):e164. doi: 10.1371/journal.pcbi.0030164 (PMC1950952; doi:10.1371/journal.pcbi.0030164)
Supplement: Supplementary file 18 [file pcbi.0030164.st015.pdf]

TABLE S15

Design Protocol		248	250	251	252	253	254	255	310	311	314	380	382	385	386	387	428	433	434	435	436	438	Identical to Native #	Identical to Native %
**NATIVE**		**K**	**T**	**L**	**M**	**I**	**S**	**R**	**H**	**Q**	**L**	**E**	**E**	**G**	**Q**	**P**	**M**	**H**	**N**	**H**	**Y**	**Q**	**21**	
**MULTI**		**+**	**M**	**+**	**+**	**+**	**+**	**+**	**E**	**K**	**Y**	**+**	**+**	**+**	**W**	**+**	**+**	**R**	**+**	**F**	**+**	**+**	**14**	**67%**
SINGLE	1ADQ	W	P	W	P	R	+	+	S	F	G	H	K	+	W	S	+	G	D	W	W	R	4	19%
	1DN2	M	V	A	+	R	R	+	Y	W	W	+	Y	R	P	F	L	W	+	F	+	H	5	24%
	1FC2	R	R	F	H	+	+	W	+	+	W	R	V	P	V	Y	Y	R	+	+	W	I	6	29%
	1FCC	+	+	G	+	R	+	K	R	W	K	+	+	W	W	K	+	+	+	R	+	+	11	52%

FC Interface Residues: columns 248–438
