# Supplementary material for: Design of Multi-Specificity in Protein Interfaces
Source: PLoS Comput Biol. 2007 Aug 24;3(8):e164. doi: 10.1371/journal.pcbi.0030164 (PMC1950952; doi:10.1371/journal.pcbi.0030164)
Supplement: Supplementary file 19 [file pcbi.0030164.st016.pdf]

TABLE S16

Design Protocol		Rac Interface Residues																					Identical to Native	
		12	13	31	32	33	34	35	36	37	38	56	57	58	61	62	63	64	66	67	70	73	#	%
**NATIVE**		**G**	**A**	**E**	**Y**	**I**	**P**	**T**	**V**	**F**	**D**	**W**	**D**	**T**	**Q**	**E**	**D**	**Y**	**R**	**L**	**L**	**P**	**21**	
**MULTI**		**+**	**G**	**+**	**+**	**E**	**+**	**H**	**+**	**+**	**+**	**+**	**H**	**D**	**+**	**D**	**+**	**+**	**+**	**+**	**+**	**W**	**14**	**67%**
SINGLE	1E96	E	W	+	P	R	G	H	W	P	R	G	G	E	R	N	P	K	G	+	P	T	2	10%
	1G4U	+	G	+	+	E	+	E	+	+	E	K	K	R	+	H	+	+	M	M	W	L	9	43%
	1HE1	+	N	Q	+	Q	+	E	I	+	+	+	K	R	+	+	+	W	+	+	R	R	11	52%
	1HH4	+	R	R	S	F	W	K	T	T	F	+	S	D	N	K	R	+	+	+	+	S	6	29%
	1I4T	L	M	N	R	R	R	L	Y	+	H	+	P	H	R	I	R	W	+	H	W	R	3	14%
