# Supplementary material for: Design of Multi-Specificity in Protein Interfaces
Source: PLoS Comput Biol. 2007 Aug 24;3(8):e164. doi: 10.1371/journal.pcbi.0030164 (PMC1950952; doi:10.1371/journal.pcbi.0030164)
Supplement: Supplementary file 20 [file pcbi.0030164.st017.pdf]

TABLE S17

Design Protocol		Ubiquitin Interface Residues																								Identical to Native	
		306	307	308	309	310	311	334	336	340	342	344	345	346	347	348	349	362	364	365	366	368	370	371	372	#	%
**NATIVE**		**K**	**T**	**L**	**T**	**G**	**K**	**E**	**I**	**Q**	**R**	**I**	**F**	**A**	**G**	**K**	**Q**	**Q**	**E**	**S**	**T**	**H**	**V**	**L**	**R**	**24**	
**MULTI**		**+**	**P**	**+**	**+**	**+**	**R**	**W**	**H**	**E**	**+**	**+**	**Q**	**D**	**+**	**+**	**K**	**W**	**+**	**+**	**S**	**R**	**+**	**N**	**+**	**12**	**50%**
SINGLE	1CMX	+	P	+	+	R	R	R	Q	R	Q	F	D	R	+	D	R	P	D	W	R	M	M	F	+	5	21%
	1FXT	H	S	M	R	+	P	W	K	G	+	+	W	G	R	P	K	A	N	L	E	W	+	W	+	5	21%
	1NBF	R	L	+	R	S	R	T	H	E	+	D	Q	R	+	+	+	D	+	T	S	R	S	I	+	7	29%
	1S1Q	P	G	R	A	Q	H	K	K	P	+	+	W	P	+	W	A	W	M	+	E	S	N	P	G	4	17%
	1WR6	Q	R	+	H	N	G	V	F	V	K	H	N	D	+	H	K	R	W	Y	V	W	I	W	K	2	8%
	1WRD	+	V	R	W	H	P	T	H	F	W	L	R	R	+	R	K	K	W	W	L	+	R	T	P	3	13%
	2D3G	+	N	I	R	S	G	G	L	E	+	+	S	H	H	G	M	I	V	K	G	+	Q	W	H	4	17%
