# Supplementary material for: Design of Multi-Specificity in Protein Interfaces
Source: PLoS Comput Biol. 2007 Aug 24;3(8):e164. doi: 10.1371/journal.pcbi.0030164 (PMC1950952; doi:10.1371/journal.pcbi.0030164)
Supplement: Supplementary file 21 [file pcbi.0030164.st018.pdf]

TABLE S18

Design Protocol		CDC42 Interface Residues																								Identical to Native	
		32	33	34	35	36	37	38	39	40	41	56	57	59	60	61	62	63	64	65	66	67	70	71	74	#	%
NATIVE		Y	V	P	T	V	F	D	N	Y	A	F	D	A	G	Q	E	D	Y	D	R	L	L	S	Q	24	
**MULTI**		**+**	**H**	**E**	**S**	**T**	**+**	**W**	**+**	**+**	**+**	**M**	**+**	**N**	**+**	**+**	**+**	**+**	**+**	**R**	**+**	**+**	**+**	**+**	**R**	**15**	**63%**
SINGLE	1DOA	I	P	R	W	S	S	W	F	W	G	Y	W	W	N	K	T	W	+	R	+	+	+	R	W	4	17%
	1GRN	W	H	E	P	Q	+	W	I	T	L	D	F	I	H	+	+	+	+	R	W	I	R	F	S	5	21%
	1GZS	+	W	E	+	E	W	R	R	H	+	M	P	K	+	+	M	M	F	N	+	+	H	+	R	8	33%
	1KI1	T	G	N	S	+	D	W	+	W	W	+	H	H	+	D	Y	R	F	P	+	+	+	M	N	7	29%
	1NF3	L	H	R	W	S	+	W	F	+	L	Y	G	K	Q	T	Y	P	K	K	W	W	+	W	M	3	13%
