# Supplementary material for: Design of Multi-Specificity in Protein Interfaces
Source: PLoS Comput Biol. 2007 Aug 24;3(8):e164. doi: 10.1371/journal.pcbi.0030164 (PMC1950952; doi:10.1371/journal.pcbi.0030164)
Supplement: Supplementary file 22 [file pcbi.0030164.st019.pdf]

TABLE S19

Design Protocol		RXR Receptor Interface Residues																									Identical to Native	
		348	352	356	373	379	390	393	394	397	398	401	413	415	416	417	419	420	421	422	423	424	426	427	430	434	#	%
**NATIVE**		**R**	**E**	**K**	**I**	**D**	**E**	**R**	**E**	**Y**	**A**	**E**	**G**	**F**	**A**	**K**	**L**	**L**	**R**	**L**	**P**	**A**	**R**	**S**	**L**	**E**	25	
**MULTI**		**H**	**R**	**+**	**T**	**+**	**+**	**+**	**+**	**+**	**M**	**+**	**M**	**+**	**P**	**R**	**+**	**+**	**+**	**H**	**+**	**S**	**M**	**M**	**H**	**+**	13	52%
SINGLE	1DKF	H	R	+	M	Q	+	I	H	W	W	+	P	+	P	R	M	R	W	N	S	S	M	L	R	+	5	20%
	1FM6	W	Q	+	V	+	W	H	+	+	Q	R	K	G	A	W	I	+	+	E	+	S	H	D	H	+	8	32%
	1MZN	H	W	R	+	+	+	M	+	+	M	R	+	P	G	+	+	E	+	H	+	E	M	F	E	R	10	40%
