# Supplementary material for: Design of Multi-Specificity in Protein Interfaces
Source: PLoS Comput Biol. 2007 Aug 24;3(8):e164. doi: 10.1371/journal.pcbi.0030164 (PMC1950952; doi:10.1371/journal.pcbi.0030164)
Supplement: Supplementary file 23 [file pcbi.0030164.st020.pdf]

TABLE S20

Design Protocol		PAPD Interface Residues																								Identical to Native	
		1	3	4	5	6	7	8	31	91	104	105	106	107	108	109	110	112	152	154	163	164	166	170	194	200	#	%
**NATIVE**		**A**	**S**	**L**	**D**	**R**	**T**	**R**	**Y**	**R**	**Q**	**I**	**A**	**L**	**Q**	**T**	**K**	**K**	**T**	**I**	**A**	**E**	**G**	**T**	**I**	**R**	**25**	
**MULTI**		**E**	**R**	**M**	**G**	**E**	**F**	**+**	**H**	**+**	**H**	**+**	**L**	**M**	**E**	**E**	**E**	**Y**	**V**	**H**	**F**	**+**	**S**	**W**	**R**	**+**	**5**	**20%**
SINGLE	1NOL	S	P	Y	F	E	R	+	W	+	+	M	L	+	F	I	W	Y	V	M	R	W	+	Y	K	K	5	20%
	1PDK	H	R	Y	R	K	+	+	H	+	W	H	W	+	K	V	+	R	V	M	W	W	M	W	R	W	4	20%
	1QPP	F	Y	M	W	H	W	G	P	W	H	R	K	W	A	+	W	Y	W	D	I	R	S	W	E	+	2	8%
