# Supplementary material for: Design of Multi-Specificity in Protein Interfaces
Source: PLoS Comput Biol. 2007 Aug 24;3(8):e164. doi: 10.1371/journal.pcbi.0030164 (PMC1950952; doi:10.1371/journal.pcbi.0030164)
Supplement: Supplementary file 24 [file pcbi.0030164.st021.pdf]

TABLE S21

PROMISCUOUS PROTEIN	DIP ID NUMBER	ORGANISM	EVALUE	DIP PROTEIN DESCRIPTION
**1 FYN SH3**	DIP:198N	*Mus musculus*	2.00E-29	Protein-tyrosine Kinase Fyn
**2 IMPORTIN BETA**	DIP:2357N	*Saccharomyces cerevisiae*	3.00E-82	KAP95 Protein
**3 OVOMUCOID INHIBITOR**	N/A	N/A	N/A	N/A
**4 CHEY**	DIP:6052N	*Escherichia coli*	8.00E-38	Chemotaxis Protein CheY
**5 THIOREDOXIN**	DIP:5552N	*Saccharomyces cerevisiae*	4.00E-12	Thioredoxin I
**6 HPR**	DIP:6180N	*Escherichia coli*	1.00E-09	Phosphocarrier protein HPr (Histidine-containing protein)
**7 IL6**	DIP:95N	*Homo sapiens*	1.00E-179	Membrane Glycoprotein GP130 Precursor
**8 BETA LACTAMASE**	N/A	N/A	N/A	N/A
**9 ELASTASE**	DIP:378N	*Sus scrofa domestica*	1.00E-132	Pancreatic Elastase I Precursor
**10 PPR**	DIP:241N	*Homo sapiens*	6.00E-97	Peroxisome Proliferator-activated Receptor
**11 RAN**	DIP:1357N	*Saccharomyces cerevisiae*	1.00E-101	GTP-binding protein GSP2
**12 RAS**	DIP:2263N	*Saccharomyces cerevisiae*	3.00E-47	GTP-binding protein RAS2
**13 ACTIN**	DIP:310N	*Saccharomyces cerevisiae*	0	actin
**14 TRANSDUCIN BETA GAM**	DIP:954N	*Saccharomyces cerevisiae*	2.00E-66	GTP-binding protein beta chain STE4
**15 FC IGG1**	N/A	N/A	N/A	N/A
**16 RAC**	DIP:862N	*Drosophila melanogaster*	1.00E-96	GTP-binding Protein Rac2
**17 UBIQUITIN**	DIP:1549N	*Saccharomyces cerevisiae*	9.00E-32	Ubiquitin
**18 CDC42**	DIP:862N	*Saccharomyces cerevisiae*	4.00E-87	Cell Division Control Protein CDC42
**19 RXR RECEPTOR**	DIP:641N	*Homo sapiens*	1.00E-138	Retinoic Acid Receptor X-alpha
**20 PAPD**	DIP:6196N	*Escherichia coli*	1.00E-120	Chaperone Protein PapD Precursor
